# Effectiveness of an integrated diabetes care package at primary healthcare facilities: a cluster randomised trial in Pakistan

**DOI:** 10.3399/bjgpopen18X101618

**Published:** 2018-12-12

**Authors:** Muhammad Amir Khan, John D Walley, Nida Khan, Joseph Hicks, Maqsood Ahmed, Shaheer Ellahi Khan, Muhammad Ahmar Khan, Haroon Jehangir Khan, Anthony D Harries

**Affiliations:** 1 Chief Coordinating Professional, Association for Social Development, Islamabad, Pakistan; 2 Professor of International Public Health, Nuffield Centre for International Health and Development, Leeds Institute of Health Sciences, Leeds, UK; 3 Project Coordinator, Association for Social Development, Islamabad, Pakistan; 4 Senior Medical Statistician, Nuffield Centre for International Health and Development, Leeds Institute of Health Sciences, Leeds, UK; 5 Senior Professional, Association for Social Development, Islamabad, Pakistan; 6 Assistant Professor, Humanities and Social Sciences Department, Bahria University, Islamabad, Pakistan; 7 Research Coordinator, Association for Social Development, Islamabad, Pakistan; 8 Director, NCD & Mental Health, Directorate General of Health Services, Lahore, Pakistan; 9 Senior Advisor, Department of Research, International Union Against Tuberculosis and Lung Disease, Winchester, UK

## Abstract

**Background:**

There were an estimated 7 million people living with diabetes in Pakistan in 2014, and this is predicted to reach 11.4 million by 2030.

**Aim:**

To assess if an integrated care package can achieve better control of diabetes.

**Design & setting:**

The pragmatic cluster randomised controlled trial (cRCT) was conducted from December 2014–June 2016 at 14 primary healthcare facilities in Sargodha district. Opportunistic screening, diagnostic testing, and patient recording processes were introduced in both the control 'testing, treating, and recording' (TTR) arm, and the intervention 'additional case management' (ACM) arm, which also included a clinical care guide and pictorial flipbook for lifestyle education, associated clinician training, and mobile phone follow-up.

**Method:**

Clinics were randomised on a 1:1 basis (sealed envelope lottery method) and 250 patients recruited in the ACM arm and 245 in the TTR-only arm (age ≥25 years and HbA1c >7%). The primary outcome was mean change in HbA1c (%) from baseline to 9-month follow-up. Patients and staff were not blinded.

**Results:**

The primary outcome was available for *n* = 238/250 (95.2%) participants in the ACM arm and *n* = 219/245 (89.4%) participants in the TTR-only arm (all clusters). Cluster level mean outcome was -2.26 pp (95% confidence intervals [CI] = -2.99 to -1.53) for the ACM arm, and -1.44 pp (95% CI = -2.34 to -0.54) for the TTR-only arm. Cluster level mean ACM–TTR difference (covariate-unadjusted) was -0.82 pp (95% CI = -1.86 to 0.21; *P* = 0.11).

**Conclusion:**

The ACM intervention in public healthcare facilities did not show a statistically significant effect on HbA1c reduction compared to the control (TTR-only) arm. Future evaluation should assess changes after a longer follow-up period, and minimal care enhancement in the comparator (control) arm.

## How this fits in

Integrated care at primary and secondary level public health facilities is an approach currently being recommended for extended coverage and continuity of diabetes care. Diabetes care includes both clinical and lifestyle modification components. In Pakistan, as most other developing countries, contextualised integrated care has never been evaluated so evidence was required to inform further measures for scaling the integrated diabetes care in low income country setting. To widen the scope of learning, a process evaluation of integrated care delivery was also conducted and published.^1^

## Introduction

There were an estimated 7 million people living with diabetes in Pakistan in 2014,^2^ with the number increasing by about 250 000 each year and predicted to reach 11.4 million by 2030.^3^ Diabetes contributes to 3% of the total deaths in Pakistan.^4^ At least 3.3 million (48%) patients with diabetes in Pakistan were undiagnosed in 2014,^5^ mostly due to obesity-related gradual adult-onset type 2 diabetes mellitus. Type 1 diabetes is a less common, severe, and rapid onset disease, requiring insulin injections and specialist care. In this study and article, ‘diabetes’ refers specifically to type 2 diabetes mellitus. Diabetes, hypertension, and the related cardiovascular diseases comprise the highest burden of non-communicable diseases (NCDs).

In Pakistan, a National Action Plan for Non-Communicable Disease Prevention and Control (NAP–NCD) was released in May 2004.^6^ This plan emphasises prevention, screening, management, monitoring, and surveillance of NCDs.^7^ However, there has been little in the way of implementation, mainly due to the lack of an intervention package for integrating diabetes prevention and control at the primary healthcare facility level. In Pakistan, rural health centres and sub-district hospitals are the primary healthcare facilities responsible for providing diagnosis, treatment, and prevention for both communicable diseases and NCDs. However, doctors and 3-year trained allied professionals (known as ‘paramedics’ in Pakistan) involved in diabetes care at this level lack standardised guidelines and/or operating practices to diagnose, treat, educate, follow-up, and report on patients with diabetes, and there are inadequate resources, such as diagnostic consumables and drugs, to support uninterrupted diabetes care; no records are kept except of the prescription, for example. Historically, diabetes and associated hyperlipidaemia would be managed by hospital specialists, but much of the population lacked access. Specialists in internal medicine, endocrinology, cardiology, ophthalmology, and renal medicine who care for patients with diabetes complications are only available at the ‘district’ level hospitals (a district in Pakistan typically has a population of 1–3 million); the sub-district level hospitals and rural health centres have general doctors who usually see patients who self-present for care. Referral may occur for seriously ill people as an emergency. Referral for specialist assessment and opinion is less common, and referral back for continuing care is even rarer; that is, links between primary healthcare centres and district hospitals are usually lacking, and the district health system is does not currently provide quality care for patients with diabetes and associated hyperlipidaemia, or complications such as strokes.

A systematic review of 11 studies with 2616 persons with diabetes reported that structured, culturally competent interventions improved patient-related outcomes such as glycaemic control, lipid profiles, and blood pressure.^8^ A systematic review in rural areas^9^ had similar findings. Another review, also from the developed country setting, found that chronic disease management together with patient interventions were effective, while those solely targeting providers were beneficial only if baseline control was poor. There are few studies or systematic reviews of diabetes care models in lower and/or middle income countries (LMICs). One review identified gaps as compared to international standards, and that most interventions focused only on the provider and not the patient.^10^ Another identified that comprehensive care models — which included collaboration, education, standardisation, resource optimisation (as in the present intervention), and technological innovation — were most successful.^11^

Based on the WHO ‘Package of Essential Non-communicable Disease Interventions for Primary Health Care’ (‘PEN’) guidance,^12^ an intervention package was developed for the delivery of quality primary health care through the district healthcare system. This package included two main components: 1) testing, treating, and recording (TTR), including the screening of overweight adults and maintenance of patient records; and 2) an additional case management (ACM) package, including standardised education on healthy lifestyle (that is, diet, exercise, and smoking cessation), standardised drug treatments for diabetes and associated hyperlipidaemia, and active clinical follow-up of patients. The package was then piloted in four primary healthcare facilities, where the feasibility of care protocols was assessed through analysis of patient records, as well as observations and interviews. Based on the findings, the details of the screening, recording and care package were refined.

In Pakistan, at the primary healthcare level, there was a lack of standard screening, testing, and recording practices, as well as case management and lifestyle education guides for NCDs. Using a cRCT, an NCD screening, testing, and recording package was implemented across all trial facilities to allow uniform recruitment and assessment of patients with diabetes. In intervention facilities, an additional case management package was then implemented, aimed at improving’ the glycaemic control and lipid profiles of patients with type 2 diabetes, and its effectiveness was evaluated.

## Method

### Study design, setting, and participants

A parallel arm, cRCT was conducted between December 2014–June 2016 in the Sargodha district in Punjab, Pakistan. In addition to a district-level secondary hospital, 14 rural health centres and 6 sub-district hospitals provide public funded care to 3.1 million population in the district (2:1 rural–urban split). After checking the in-place basic services (such as laboratory) at each eligible site with the district health office, nine rural health centres, and five sub-district hospitals (total *n* = 14) were recruited and randomised into the trial. In each of the 14 selected clusters, communal consent for the catchment population's participation in the trial was taken from the community leaders who were identified and invited with the help of staff at respective health facility. These community leaders represented a) religious leaders, b) political leaders (local woman councillor), c) health representative of union council, d) school teachers, e) local press reporters, and f) local businessmen. The study involves only the outpatient department of facilities, which have general practice doctors seeing un-referred primary care patients. A cluster design was used because it would not have been feasible to expect providers to effectively apply or withhold many of the intervention components based on a patient’s treatment allocation (for example, standardised diagnosis), and because of the risk of contamination between patients.

Patients were eligible if they were understood by the facility doctor to be newly diagnosed with diabetes (fasting blood glucose ≥126; random blood glucose ≥200), aged ≥25 years, not pregnant, and not likely to move away from their initial catchment area during follow-up. The authors attempted to recruit all eligible patients attending the selected public facilities between December 2014–September 2015. Informed consent was obtained from patients using a standard consent form administered by an allied professional (paramedic) at the respective public healthcare facility.

### Randomisation and blinding

The 14 facilities were randomised to the TTR-only or the ACM arm on a 1:1 basis using a lottery method with sealed envelopes. In the presence of a five member committee, a staff member from the provincial directorate randomly selected seven envelopes for each arm from among 14 sealed envelopes, each containing a recruited facility name. The committee consisted of the Director Non-Communicable diseases in Punjab, two representatives from the Directorate General of Health Services Punjab, and two Association for Social Development representatives. It was not possible to blind healthcare providers or patients to the treatment allocation due to the nature of the trial procedures, such as provider training and patient education. However, the laboratory staff who carried out the testing for outcomes were blinded to the cluster allocation.

### Procedures

In the TTR-only arm facilities, the existing situation meant that there was training on screening, diagnosis, testing, and recording, but no operational guidelines available for doctors or allied professionals on the additional case management of diabetes and related high cholesterol. Clinicians therefore managed such conditions according to their existing knowledge and practices, and without reference to any specific guidelines and/or tools. The district health office was requested to make the same drugs available at all facilities, through the routine district drug system, though with some top up in the ACM facilities. For this study, the interventions/ resources provided in both arms were over and above usual care, and included: a) equipment and supplies for patient screening at the first visit, including weight and height scales, glucometers and glucose strips, and electronic sphygmomanometers; b) staff training on patient screening and diagnosis at the first visit; and c) a patient card for recording core biomedical measures at baseline and follow-ups. Patients were also given vouchers so that they could get glycated haemoglobin (HbA1c) and serum cholesterol measured at no cost at specified laboratories. All these TTR inputs were the same for both arms.

**Figure 1.**
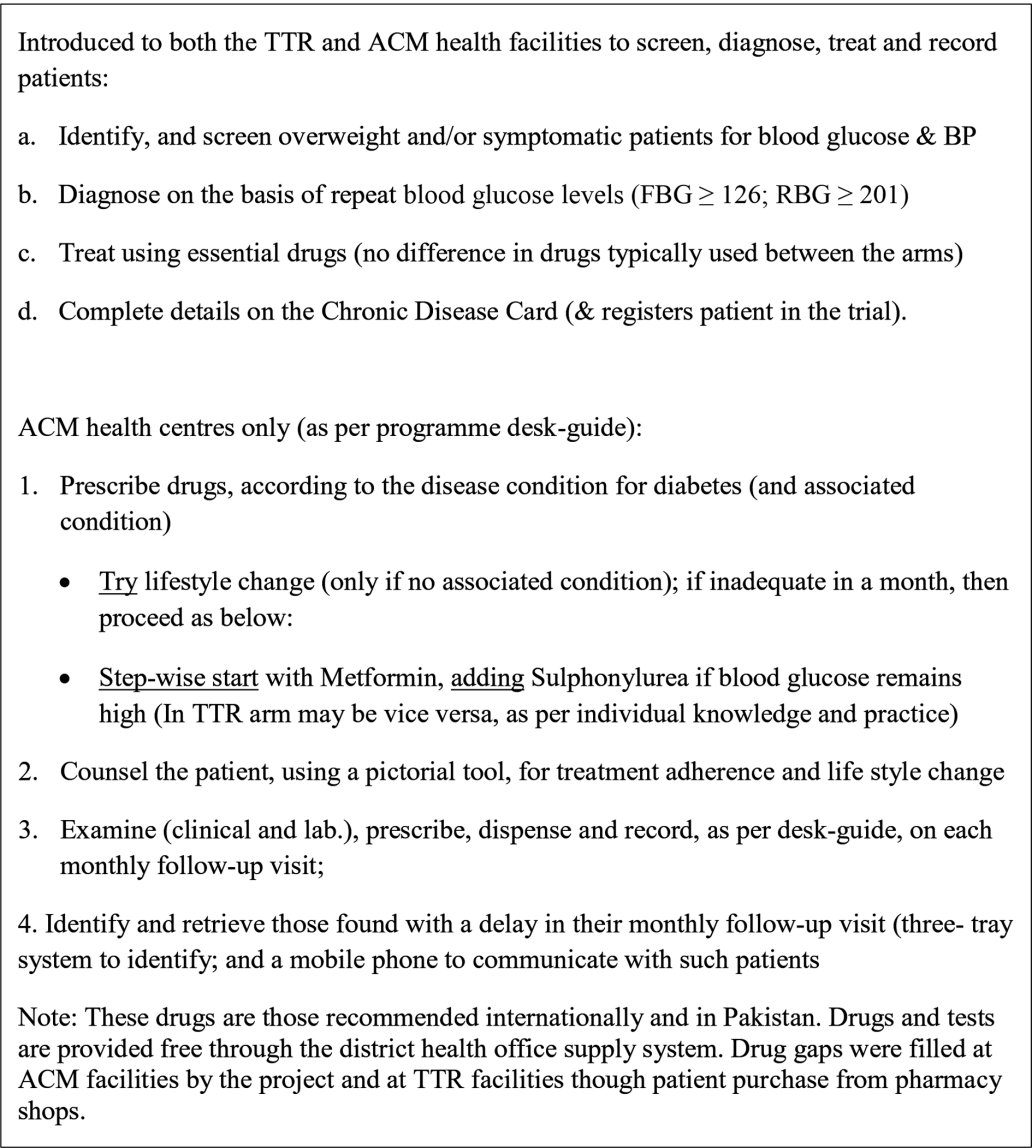
Diabetes intervention (ACM) and control (TTR-only) care package details. ACM = additional case management. BP = blood pressure. FBG = fasting blood glucose. RBG = random blood glucose. TTR = testing, treating, and recording.

In the ACM arm, in addition to the TTR inputs above, all facilities received additional case management ACM enhancements including: a) a context-adapted desk guide for step-by-step management of diabetes and associated high cholesterol, and information about referral of severe or complex cases; b) patient education on diabetes care and lifestyle change, using contextualised communication materials; and c) monthly appointment review and reminders (through SMS text messaging). A more detailed description of the TTR and ACM procedures is provided in Figure 1 .

Two key diabetes drugs, metformin and sulphonylurea, were available both in the TTR-only and ACM arm facilities, mainly through the respective district health office. However, the ACM arm desk-guide included a step-by-step approach to adding drugs and amending dosage of diabetes drugs, as well as recommending a statin if patient had raised cholesterol. Further details are included in the process evaluation study published alongside the results of this trial,^1^ and the guides and tools are available elsewhere.^13^

### Data collection and outcomes

The inclusion of patients happened at an expected pace (as informed by piloting experience), although this pace varied slightly across facilities. All data were recorded on patients’ chronic disease cards. At both sub-district hospitals and rural health centres, the facility doctor recorded the clinical data (for example, diagnosis, prescription, and blood pressure) and the allied professional recorded the nonclinical data (such as age and weight) at diagnosis and at eight subsequent monthly follow-up visits. The doctor, using electronic sphygmomanometer during clinical consultation, checked and recorded the blood pressure. A designated allied staff member at the facility checked and recorded the fasting and/or random blood glucose levels using a glucometer and strips. A designated non-public laboratory collected samples and tested for HbA1cand serum cholesterol levels, and an allied staff member at the facility noted the results on the patient's card. Data were then transferred from patient record cards and entered into an SPSS database. The research team tried to contact patients who did not return for their 9-month follow-up appointment for 3 months before they were classed as lost to follow-up.

All outcomes were recorded at the patient level. The primary outcome was the percentage point change in HbA1c, the internationally recognised standard test (of glucose control over the previous 3 months), between baseline and 9 months. Secondary outcomes were the change between baseline and 9 month in systolic blood pressure (mmHg), diastolic blood pressure (mmHg), and total serum cholesterol (mg/dL), and 9-month glycaemic control (HbA1c <7%) and hypertension control (systolic blood pressure <140 mmHg).

### Statistical analysis

Data were single entered into SPSS (version 17). To minimise data errors, data quality assurance procedures were used, including training of data entry operators and checking data entry quality at regular intervals.^14^

All data were analysed according to the original allocation of clusters, but patients with missing outcome and/or covariate data were excluded as necessary (that is, depending on whether the data were required for the analysis). Cluster-level methods of analysis were used that were suitable for cRCTs with relatively few clusters per arm,^15^ producing crude and covariate-adjusted results. All adjusted analyses controlled for patient sex, age, number of schooling years, baseline BMI, and smoking status, as well as relevant baseline outcomes. All analyses exclude patients’ missing outcome and/or covariate data, as required by the relevant analysis. In addition, change in HbA1c and glycaemic control analyses were also adjusted for baseline hypertension status, cholesterol level, and HbA1c level. Change in systolic blood pressure and hypertension control analyses were also adjusted for baseline cholesterol level and systolic blood pressure. Change in diastolic blood pressure analysis was also adjusted for baseline cholesterol level and diastolic blood pressure. Change in cholesterol analysis was also adjusted for baseline cholesterol and hypertension status

Statistical significance was set at the 5% level, and two-sided *P* values were calculated. More details on statistical analyses are available from the author on request.

### Sample size calculation

For the primary outcome, the authors aimed to be able to detect a ≥0.5 percentage point decrease in HbA1c from baseline to 9 months in the ACM arm compared to the TTR-only arm. A standard deviation of 2% was assumed,^16^ as well as an intracluster correlation coefficient of 0.018,^17^ and a 10% loss to follow-up rate. A significance level of 5% was set. To detect the minimum clinically important difference of 0.5 percentage points between the mean change (baseline to follow-up) in HbA1c for the ACM arm compared to the TTR-only arm with 80% power required a total sample size of 448 patients across 14 facilities. The research team therefore aimed to recruit an average of 32 patients per cluster over 6 months.

## Results

**Figure 2.**
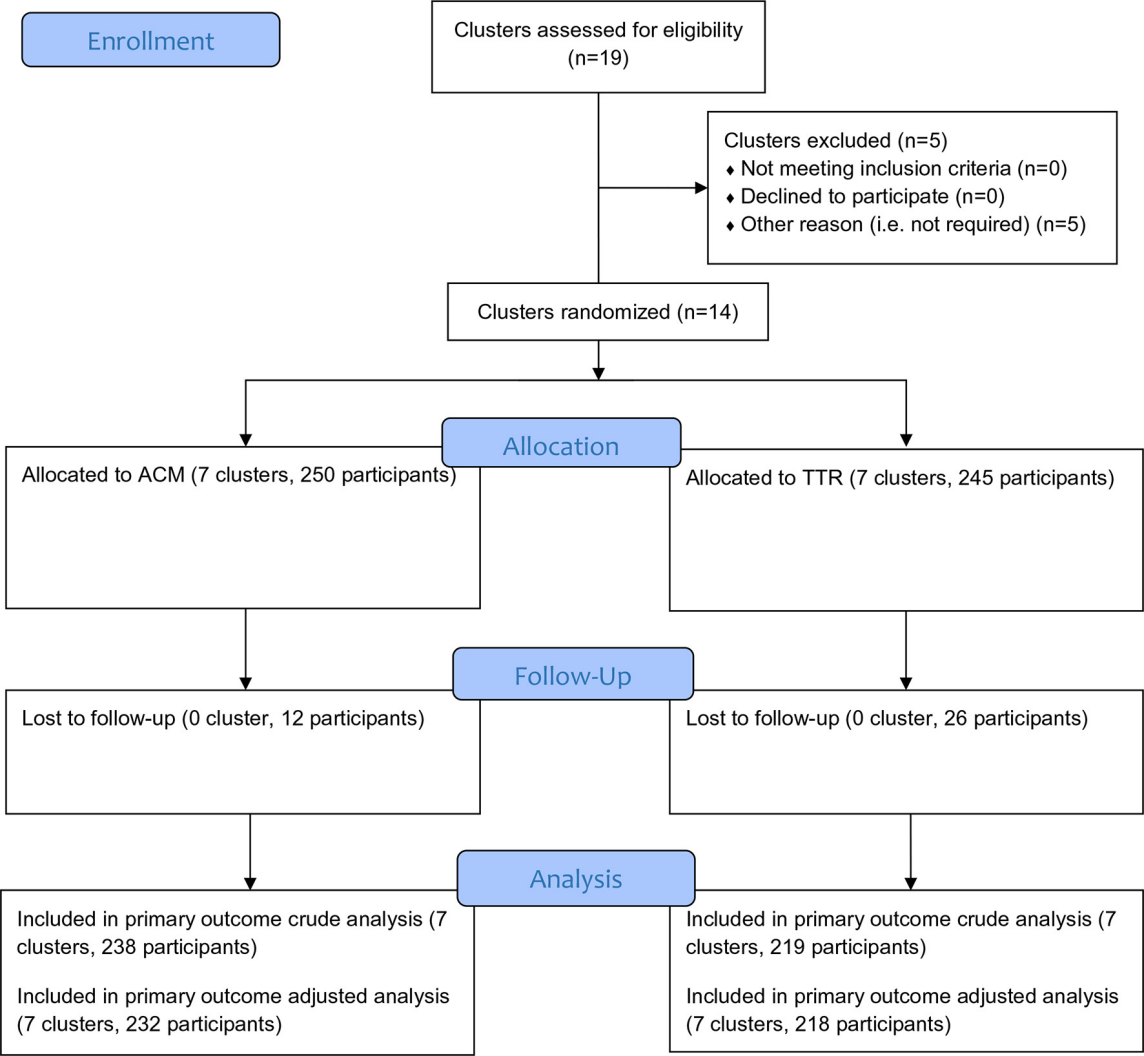
CONSORT trial flow chart ACM = additional case management. TTR = testing, treating, and recording.

Patients were recruited in the trial during the period August 2014–September 2015; and the follow-up and outcome measurements were completed by the end of July 2016. Figure 2 shows the trial design flowchart. Baseline characteristics of trial patients are shown in Table 1. There were no substantial differences in patient characteristics between the arms.

**Table 1.** Baseline characteristics of clusters and patients

Characteristics	ACM, *n* (%)	TTR-only, *n* (%)
**Clusters**		
Total	7 (100.0)	7 (100.0)
**Doctors**		
Male	7 (100.0)	7 (100.0)
Female	0 (0.0)	0 (0.0)
**Paramedics**		
Male	5 (71.4)	3 (42.9)
Female	2 (28.6)	4 (57.1)
**Participants in clusters**		
Total	250 (50.5)	245 (49.5)
Mean cluster size (SD)	35.7 (± 10.4)	35 (± 15.4)
**Sex**		
Male	92 (36.8)	97 (39.6)
Female	158 (63.2)	148 (60.4)
Mean age, years (SD)	46.1 (± 10.4)	46.1 (± 9.7)
Mean education ,years (SD)	3.5 (± 4.4)	4.9 (± 4.8)
Mean BMI, kg/m^2^ (SD)	31.9 (± 7.8)	32.8 (± 8.1)
Hypertensive	60 (24.0)	68 (27.8)
Mean fasting blood sugar, mg/dL (SD)	216.3 (± 56.5)	203.8 (± 52.7)
Mean random blood sugar, mg/dL (SD)	303.3 (± 81.6)	292.7 (± 69.5)
Mean HbA1c% (SD)	10.4 (± 2.4)	10.0 (± 2.4)
Mean systolic blood pressure, mmHg (SD)	129.9 (± 16.6)	130.1 (± 17.6)
Mean diastolic blood pressure, mmHg (SD)	84.7 (± 9.4)	87.0 (± 13.3)
Mean total serum cholesterol, mg/dL (SD)	192.6 (± 51.8)	193.0 (± 46.6)

'Hypertensive' defined as baseline systolic blood pressure >140 mmHg.

ACM = additional case management. BMI = body mass index. TTR = testing, treating, and recording

There was no statistically significant difference in the baseline to 9-month follow-up change in HbA1c between the ACM and the TTR-only arms, although there was a clear reduction in HbA1c between baseline and follow-up within both arms (see Table 2). There were also no statistically significant treatment effects for any of the secondary outcomes, as shown in Table 2. These results were consistent between the crude and covariate-adjusted analyses. For all crude analyses, except for the adherence and cholesterol outcomes, data were available for 238/250 (95.2%) participants in the ACM arm and for 219/245 (89.4%) participants in the TTR arm. For the adherence outcome, data were available for 250/250 (100%) participants in the ACM arm and 245/245 (100%) participants in the TTR arm, and for the cholesterol outcome data were available for 234/250 (93.6%) participants in the ACM arm and 219/245 (89.4%) participants in the TTR arm. For all adjusted analyses, except for the adherence and cholesterol outcomes, data were available for 232/250 (92.8%) participants in the ACM arm and 218/245 (89%) participants in the TTR arm. For the adjusted analysis of the adherence outcome, data were available for 243/250 (97.2%) participants in the ACM arm 228/245 (93.1%) participants in the TTR arm, and for the cholesterol outcome data were available for 228/250 (91.2%) participants in the ACM arm and 218/245 (89%) participants in the TTR arm. The intracluster correlation coefficient for the primary outcome was 0.046 (95% CI = 0.01 to 0.149) across both arms, 0.026 (95% CI = -0.007 to 0.199) for the ACM arm and 0.044 (95% CI = -0.001 to 0.26) for the TTR alone arm. There was also no evidence for any effect modification of a possible treatment effect on the primary outcome by sex (further information available from the author on request).

**Table 2.** Primary and secondary outcome results

	ACM arm (clusters, *n* = 7)	TTR-only arm (clusters, *n* = 7)	Crude ACM-TTR difference (95% CI); *P* value^a^	Adjusted ACM-TTR difference (95% CI); *P* value^a^
**Mean outcome (95% CI)^b^**				
**Primary outcome**				
Change in HbA1c (pp)^c^	-2.26pp (-2.99 to -1.53)	-1.44pp (-2.34 to -0.54)	-0.82pp (-1.86 to 0.21); 0.11	-0.57pp (-1.44 to 0.29); 0.17
**Secondary outcomes**				
Adherence^d^	75.64% (45.56 to 105.71%)	36.29% (3.9 to 68.67%)	39.35pp (-0.02 to 78.73); 0.0501	36.06pp (-0.78 to 72.9); 0.054
Glycaemic control^e^	45.04% (32.48 to 57.6%)	34.86% (16.15 to 53.57%)	10.18pp (-10.21 to 30.57); 0.29	10.87pp (-7.92 to 29.67); 0.23
Change in SBP (mmHg)^c^	2.33 (-0.1 to 4.76)	1.47 (-4.32 to 7.26)	0.86 (-5.05 to 6.77); 0.75	1.3 (-3.41 to 6.01); 0.56
Change in DBP (mm Hg)^c^	2.81 (-1.04 to 6.66)	0.68 (-4.34 to 5.69)	2.13 (-3.54 to 7.81); 0.43	0.29 (-1.93 to 2.51); 0.78
Hypertension control^f^	73.2% (61.92 to 84.49%)	72.47% (59.36 to 85.58%)	0.73pp (-14.71 to 16.17); 0.92	0.0pp (-12 to 12.95); 0.93
Change in total serum cholesterol (mg/dL)^c^	-5.24 (-19.82 to 9.34)	2.75 (-5.73 to 11.22)	-7.99 (-23.43 to 7.45); 0.27	-5.79 (-20.22 to 8.65); 0.4

ACM = additional case management. CI = confidence intervals. TTR = test, treat, and record. PP = percentage points.

^a^All ACM–TTR differences (that is, ACM effect size measures) are based on analysis of cluster-level (mean/proportion) outcomes. ^b^Mean outcomes and their 95% confidence intervals are based on cluster-level (mean/proportion) raw outcome data. ^c^All change outcomes are calculated as outcome at 9 months minus outcome at baseline. ^d^Adherence defined as attendance of ≥4 follow-up appointments within 9 months. ^e^Glycaemic control defined as 9-month HbA1c (%) <7%. ^f^Hypertension control defined as 9-month systolic blood pressure ≤140 mmHg.

All analyses exclude patients’ missing outcome and/or covariate data as required by the relevant analysis.

The adherence to monthly follow-up — that is, ≥4 visits out of the 8 monthly follow-up visits recommended — was 75.6% for the ACM arm (which included measures such as SMS messaging to improve attendance) and 30.2% for the TTR only arm. The mean difference in adherence between two arms was 45.4% (95% CI = 38% to 53%; *P =* 0.001).

## Discussion

### Summary

There was no statistically significant difference between the ACM and TTR-only arms for the primary outcome of change in HbA1c. This lack of difference in blood glucose control might have occurred because of the following two limitations: first, the TTR-only (control) arm was more effective than existing routine care. However, to have comparable clients enrolled and outcomes recorded, training and equipment was provided and drug supply also facilitated in the TTR-only arm. This is very likely to have led to a better level of care in the TTR-only facilities than would normally be encountered in usual care practice. As a result, outcome measures in the TTR-only arm facilities, such as HbA1c and serum cholesterol, might have been better than those usually seen in routine care. In TTR-only patients, the HbA1c decrease at 9 months was -1.44 pp (95% CI = -2.34 to -0.54), and -2.26 pp (95% CI = -2.99 to -1.53) for the ACM arm patients. These are likely to be clinically significant reductions as the risk of diabetes complications drops by 35% for every percentage point decrease in HbA1c.^18^ In short, an unanticipated positive effect of the TTR on the primary outcome may have been a key reason for the trial not showing a statistically significant difference between the arms. Clearly, initial assumptions around the expected effect size from the intervention were not correct, which may be primarily due to the unanticipatedly large, positive effect of the TTR intervention. Therefore, assuming a smaller effect size and using a larger sample size may have resulted in the detection of a statistically significant intervention effect.

Second, the 9-month follow-up duration might have not been enough for the ACM intervention to achieve the desired reduction in blood glucose of diabetic patients. The ACM intervention starts with a minimum level of treatment and then gradually becomes more rigorous, adding drugs and increasing dosage during monthly follow-up visits, as needed. This gradual enhancement might have contributed to relatively slow progress in glucose control in the ACM intervention, leading to a lack of significant difference between TTR-only and ACM facilities at 9-month follow-up in the trial.

### Strengths and limitations

The main strength of this trial is that it was conducted within the routine health care using the same staff and essential drugs. It was designed and developed to be potentially replicable and sustainable in the routine system, and the intervention and research protocols and tools were piloted before the trial. However, healthcare providers and patients were not blinded to the treatment arm allocation; in addition, the trial was not meant to assess changes in diet or exercise.

### Comparison with existing literature

A randomised controlled trial evaluating the addition of a structured education manual for general practice nurses in a high income context (the UK) found no differences in HbA1c between enhanced care (ACM) and current practice (TTR-only) arms.^19^ However, this trial's ACM package was more than just the provision of education materials for healthcare providers, and health services context was also different since Pakistan is a low income setting. In the United Arab Emirates, a standard package of care that included education of healthcare professionals and patients, support for diabetes care, and improved recording of clinical information^20^ showed improvements in some but not all processes of quality care, as in the present trial. A systematic review has also found that a package of complex interventions, rather than a single intervention, resulted in better processes of care and enhanced^21^ patient outcomes,^22^ and a systematic review in rural areas^9^ had similar findings. Another review, from a developed country setting, found that chronic disease management together with patient interventions were effective, while those solely targeting providers were beneficial only if baseline control was poor, as was the case in the present trial. A review of diabetes care models in LMICs identified gaps in guidelines compared to international standards, and that most focused on the provider and not the patient; the present intervention focused on both.^10^ Another identified that comprehensive care — incorporating collaboration, education, standardisation, resource optimisation (as in the present intervention), and technological innovation — was a common feature of successful models.^11^

The present trial showed better patient adherence to the monthly follow-up visits to be examined and collect free of cost drugs. The enhanced patient education, SMS messaging, and telephone reminder components of the ACM intervention are likely to have contributed to improved adherence to the monthly follow-up. Mobile phones have also been used to ensure regular follow-up of patients in rural areas of Pakistan, and this has been shown to be helpful in lowering and normalising HbA1c levels.^23^ The reminder letters also improved patient attendance and retention for diabetes and hypertension management in rural health districts in Cameroon.^24^

### Implications for research

Future evaluation of ‘step by step’ integrated care may better be assessed after a period of follow-up exceeding 9 months. Also, a further study to separately evaluate the effectiveness of the two components of an intervention (that is, clinical care and lifestyle modification) would further add to current knowledge.

This pragmatic randomised controlled trial at public healthcare facilities in Pakistan did not show a statistically significant difference in HbA1c between intervention and control arms (although clinically relevant reductions were found within both arms between baseline and endline). The strengthening of care in the control arm (above routine care) and relatively short duration of follow-up (of 9 months) prior to the outcome measurement cannot be ruled out as plausible explanations for the lack of treatment effect. However, patient adherence to monthly follow-up visits was found to have improved in the intervention arm compared to the control arm.
